# Transcriptional patterns, biomarkers and pathways characterizing nasopharyngeal carcinoma of Southern China

**DOI:** 10.1186/1479-5876-6-32

**Published:** 2008-06-20

**Authors:** Weiyi Fang, Xin Li, Qingping Jiang, Zhen Liu, Huiling Yang, Shuang Wang, Siming Xie, Qiuzhen Liu, Tengfei Liu, Jing Huang, Weibing Xie, Zuguo Li, Yingdong Zhao, Ena Wang, Francesco M Marincola, Kaitai Yao

**Affiliations:** 1 Cancer Research Institute of Southern Medical University, Key Lab for Transcriptomics and Proteomics of Human Fatal Diseases Supported by Ministry of Education and Guangdong Province, 510515, PR China; 2 Infectious Disease and Immunogenetics Section, Department of Transfusion Medicine, Warren G. Magnuson Clinical Center, Bethesda, MD 20892, USA; 3 Biometrics Research Branch, National Cancer Institute, Bethesda, MD 20892, USA; 4 Medical Clinical Research Institute, The first affiliated hospital of Nanhua University, Hengyang City, Hunan Province, 421001, PR China

## Abstract

**Background:**

The pathogenesis of nasopharyngeal carcinoma (NPC) is a complicated process involving genetic predisposition, Epstein-Bar Virus infection, and genetic alterations. Although some oncogenes and tumor suppressor genes have been previously reported in NPC, a complete understanding of the pathogenesis of NPC in the context of global gene expression, transcriptional pathways and biomarker assessment remains to be elucidated.

**Methods:**

Total RNA from 32 pathologically-confirmed cases of poorly-differentiated NPC was divided into pools inclusive of four consecutive specimens and each pool (T1 to T8) was co-hybridized with pooled RNA from 24 normal non-cancerous nasopharyngeal tissues (NP) to a human 8K cDNA array platform. The reliability of microarray data was validated for selected genes by semi-quantitative RT-PCR and immunohistochemistry.

**Results:**

Stringent statistical filtering parameters identified 435 genes to be up-regulated and 257 genes to be down-regulated in NPC compared to NP. Seven up-regulated genes including CYC1, MIF, LAMB3, TUBB2, UBE2C and TRAP1 had been previously proposed as candidate common cancer biomarkers based on a previous extensive comparison among various cancers and normal tissues which did not, however, include NPC or NP. In addition, nine known oncogenes and tumor suppressor genes, MIF, BIRC5, PTTG1, ATM, FOXO1A, TGFBR2, PRKAR1A, KLF5 and PDCD4 were identified through the microarray literature-based annotation search engine MILANO, suggesting these genes may be specifically involved in the promotion of the malignant conversion of nasopharyngeal epithelium. Finally, we found that these differentially expressed genes were involved in apoptosis, MAPK, VEGF and B cell receptor signaling pathways and other functions associated with cell growth, signal transduction and immune system activation.

**Conclusion:**

This study identified potential candidate biomarkers, oncogenes/tumor suppressor genes involved in several pathways relevant to the oncogenesis of NPC. This information may facilitate the determination of diagnostic and therapeutic targets for NPC as well as provide insights about the molecular pathogenesis of NPC.

## Background

The synergetic effect of virus infection, genetic aberrations and environmental factors may lead to sequential alterations of gene expression involved in several biological pathways at different stages of nasopharyngeal carcinoma (NPC) oncogenesis. Contemporary advances in cancer genomic analysis including microarray, array-based high throughput comparative genomic hybridization (aCGH), detection of promoter hypermethylation, and analysis of gene mutation have greatly accelerated our understanding of NPC-associated genes. With the increased application of microarray technology to investigate genes differentially expressed in NPC[1,2], many functional associations with NPC pathogenesis have been gradually discovered[3,4]. Accumulation of CGH data indicated that genetic imbalances occur consistently in particular chromosomal regions in which a high frequency of oncogenes and tumor suppressor genes are gathered [3-8]. However, in spite of these important insights the pathogenesis of NPC remains elusive as a complete identification of genes associated with its development is not available. Highly frequent mutations of p53 gene, a classical tumor suppressor gene, associated with most of human malignancies, do not link to the pathogenesis of sporadic NPC consistently, strongly suggesting NPC has its specific pattern of gene expression and other genes may play more significant roles in its oncogenesis and tumor progression [9]. Therefore, in the present study, we utilized 8K cDNA microarray and several bioinformatics tools (KEGG database, online MILANO, BRB arraytool's gene set comparison) to profile differential gene expression between NPC and NP samples from Southern China, the region with highest NPC prevalence in the world. Several oncogenes and tumor suppressor genes were identified as candidate biomarkers associated with important pathways relevant to NPC oncogenesis, this may facilitate the development of important diagnostic and therapeutic targets for NPC as well as provide further insights about the molecular pathogenesis of NPC.

## Methods

### Samples collection and screening

One-hundred-and-two primary tumor biopsies diagnosed as poorly differentiated squamous cell carcinoma were obtained from primary NPC patients. In addition, 24 non-cancer nasopharyngeal (NP) tissues were obtained from patients with or without NPC. Biopsy samples containing more than 70% of tumor cells [10] were selected for further analysis. All participants (with/without NPC) gave their informed consents before the biopsies at Jiangmen Center Hospital, Guangdong Province and Tumor Hospital of Hunan Province. In addition, three well-characterized NPC cell lines, 5–8F with highly tumorigenic and metastatic potential and 6–10B and CNE2 with tumorigenic potential but disability to metastasize were collected and analyzed.

### Hybridization to arrays

All experiments were performed in Shenzhen Chipscreen Biosciences Limited of China [11]. A pooling strategy was applied; every four NPC biopsies were pooled and three cell lines (5–8F, 6–10B and CNE2) were pooled (1:1:1). In addition, all normal NPs were pooled together to be used as the normal reference. Total RNA samples extracted by Trizol reagent were further purified using Qiagen RNeasy mini kit (Qiagen, Inc.). 20 μg of total RNA samples isolated respectively from each NPC pool and corresponding normal reference were labeled by Cy5-dCTP and Cy3-dCTP respectively in the presence of 2 μg oligo(dT)18 primer in a reverse transcription reaction. The resulting labeling reactions were treated with 2 μl of 0.5 M NaOH hydrolyzing RNA for 15 mins at room temperature, and then neutralized with 2 μl of 0.5 M HCl. The labeled first-strand products were purified by QIAquick PCR purification kit and dried by speedvac. Finally, the balanced mixture of Cy5- and Cy3-labeled targets was co-hybridized against the 8K Human cDNA microarrays (CSC-GE) from Shenzhen Chipscreen Biosciences Limited, in a humidified chamber in 30 μl of hybridization solution (7.5 μl of 4 × hybridization buffer solution, 15 μl of 50% formamide and 7.5 μl of purified water) at 42°C overnight after denaturized at 95°C for 5 minutes. Slides were washed twice for 20 min each time in 0.1% SSC at 55°C., dried, and scanned with a Generation III array scanner (Amersham Pharmacia). The scanned images were converted to digital data by Arrayvision 6.0.

### Statistical analysis and bioinformatics analysis

Statistical identification of candidate NPC biomarkers was based on BRB Array tool version 3.6[12]; the raw data were first filtered to exclude uninformative spots using the following filtering parameters: 1) minimum intensity was set 200 in both fluorescence channels; 2) the lowess smother correction method with median over entire array was used for normalization; 3) More than 60% of expression data should have at least a 2-fold change in either direction from gene's median value; 4) Percent of data missing or filtered out could not exceed 50%. Class comparison between groups of arrays was performed by paired samples (NPC and NP from same array) with a univariate significance threshold set at a p_2_-value < 0.005.

To computationally analyze KEGG pathways based on differential gene expression data, Principal Component Analysis was performed by using BRB array tool. Gene set expression comparisons were performed at a nominal 0.005 level of the Hotelling T-square test. P-values of the univariate test were calculated, followed by 1000 multivariate permutations test. Significant pathways that have more genes differentially expressed between NPC and NP classes than expected by chance were selected [13].

Genes differentially expressed by NPC compared to NP samples were evaluated for functional annotations according to the Microarray literature-based annotation(MILANO)[14,15]. This program performed automatic searches in Pubmed collection for articles containing co-occurrences of search terms. A list of genes was used by pasting differentially expressed genes in the "Primary Search Term" field; "oncogene, proto-oncogene, tumor suppressor gene and nasopharyngeal carcinoma" search terms where targeted within the "Secondary Search Term" field. The output was a table containing the number of hits within the Pubmed literatures for each pair of search terms.

### Semiquantitative RT-PCR and Immunohistochemistry

Purified total RNA was treated with RNase-free DNase I (TaKaRa). After removal of the DNase I, cDNA was reversely transcribed from 1 μg of total RNA using oligo(dT)18 in 20 μl reaction volume. Five randomly selected genes (PDGFRA, BIRC5, CTGF, EBI2, and TGFBR2) differentially expressed in NPC samples compared with NP samples according to our microarray data and an invariant housekeeping gene control, ACTG1 (actin, gamma 1), were amplified from 5% of synthesized cDNA according to the designed primers. The primer pair designed for each gene spanned at least an intron to distinguish possibly amplified cDNA products from genomic DNA. Subsequently, 5 μl of each PCR reaction product was analyzed on 1.5% agarose gel from which the intensity of each band was quantitated by the Vilber gel documentation system (Vilber Limited). The RT-PCR signal from each gene was normalized by the ACTG1 gene.

Paraffin sections (4 μm) available from NPC and NP samples were deparaffinized in 100% xylene and re-hydrated in descending ethanol series according to standard protocols. Microwave-induced epitope retrieval was performed in 10 mM citrate buffer at 95°C for 15 min. Endogenous peroxidase activity and non-specific antigen were blocked with peroxidase blocking reagent containing 3% hydrogen peroxide and serum followed by incubation with rabbit anti- BIRC5, CTGF and TGFBR2 protein antibody (1:100, Boshide Company, China) at 4°C overnight. After washing, the sections incubated using biotin-goat anti-mouse/rabbit at room temperature for 10 minutes, were then conjugated with horseradish peroxidase (Maixin Company, China). The peroxidase reaction was developed with 3, 3-diaminobenzidine chromogen solution in DAB buffer substrate. Sections were counterstained with hematoxylin, mounted in neutral gum and analyzed using a bright field microscope. The results were finally analyzed by Mann-Whitney test of SPSS11.5 software.

## Results

### NPC samples screening

One-hundred-and-two primary NPC samples were screened to ascertain the validity of the diagnosis based on frozen sections. Among them, 32 samples containing more than 70% of cancer cells [10] were qualified for further analysis. All samples included were diagnosed as poorly differentiated NPC.

### Differentially expressed genes by NPC compare with the normal tissue

**Figure 1:**
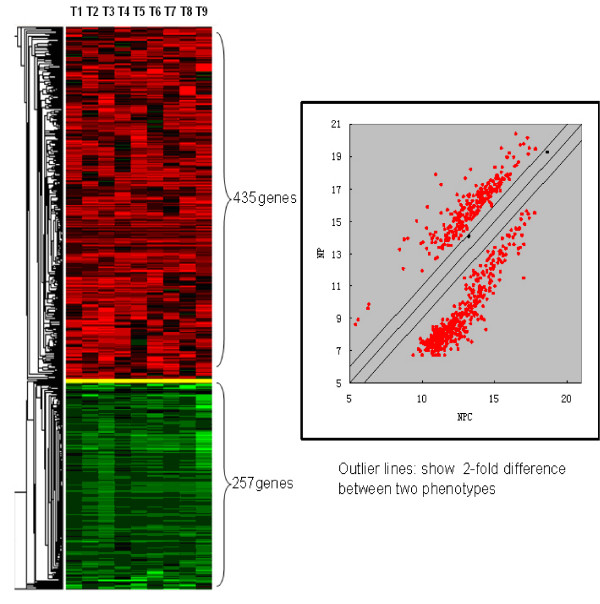
**Class comparison and hierarchical clustering analysis**. T1-T8: Pooled NPCs compared to pooled NPs; T9: Pooled NPC cells compared to pooled NPs; Green region: down-regulated genes, Red region: up-regulated genes.

All 32 qualified NPC samples were pooled into 8 pools (n = 32/4) ranging from T1 to T8 which were then co-hybridized with NP (pooled normal reference). In parallel, three NPC cell lines were pooled as T9 and co-hybridized with the NP reference as well. A Total of 692 genes were identified to be differentially expressed. Among them, 435 genes were shown to be up-regulated and 257 genes down-regulated in NPC (Table 1 and Figure 1). The top 70 up-regulated and down-regulated genes are shown in Table 2 and Table 3 respectively.

**Table 1:** Differentially expressed genes between NPC and NP

**Differential expression**	**Number of genes**	**Fold difference (NPC/NP)**
Up-regulated	435	2.8–70.3
Down-regulated	257	0.01–0.46
Total	692	

Number of genes significant at 0.005 level of the univariate test: 692

Global test: probability of getting at least 692 genes significant by chance (at the 0.005 level) if there are no real differences between the classes: 0.00391

**Table 2:** Top 70 up-regulated genes

**No**.	**Gene symbol**	**False discovery rate**	**Fold difference**	**Description**
1	*SCAM-1*	< 1e-07	22.68	vinexin beta (SH3-containing adaptor molecule-1)
2	*SNTB1*	< 1e-07	22.04	syntrophin, beta 1 (dystrophin-associated protein A1, 59 kDa, basic component 1)
3	*DMWD*	< 1e-07	21.75	dystrophia myotonica-containing WD repeat motif
4	*NPFF*	< 1e-07	20.72	neuropeptide FF-amide peptide precursor
5	*FXYD3*	< 1e-07	6.97	FXYD domain containing ion transport regulator 3
6	*ATP6V0B*	< 1e-07	5.77	ATPase, H+ transporting, lysosomal 21 kDa, V0 subunit c"
7	*PPIB*	< 1e-07	4.64	peptidylprolyl isomerase B (cyclophilin B)
8	*HIST1H4C*	< 1e-07	3.81	histone 1, H4c
9	*PHB*	9.40e-06	7.07	prohibitin
10	*NME1*	9.40e-06	5.00	non-metastatic cells 1, protein (NM23A) expressed in
11	*NDUFA9*	9.40e-06	3.34	NADH dehydrogenase (ubiquinone) 1 alpha subcomplex, 9, 39 kDa
12	*BUB1B*	1.20e-05	2.77	BUB1 budding uninhibited by benzimidazoles 1 homolog beta (yeast)
13	*GRK5*	1.34e-05	21.03	G protein-coupled receptor kinase 5
14	*TPI1*	1.34e-05	10.82	triosephosphate isomerase 1
15	CYC1 *	1.34e-05	10.36	cytochrome c-1
16	*GPC5*	1.55e-05	19.28	glypican 5
17	*BYSL*	1.67e-05	41.59	bystin-like
18	*COX5B*	1.67e-05	12.52	cytochrome c oxidase subunit Vb
19	*KATNB1*	1.84e-05	18.37	katanin p80 (WD repeat containing) subunit B 1
20	*PRKAB2*	2.03e-05	14.24	protein kinase, AMP-activated, beta 2 non-catalytic subunit
21	*PSMB3*	2.03e-05	7.43	proteasome (prosome, macropain) subunit, beta type, 3
22	*PET112L*	2.16e-05	21.46	PET112-like (yeast)
23	*PSMD8*	2.16e-05	4.88	proteasome (prosome, macropain) 26S subunit, non-ATPase, 8
24	*CSF1*	2.25e-05	20.35	colony stimulating factor 1 (macrophage)
25	*SFTPA2*	2.25e-05	17.66	surfactant, pulmonary-associated protein A2
26	*PTCD1*	2.25e-05	7.36	pentatricopeptide repeat domain 1
27	*AARS*	2.25e-05	6.65	alanyl-tRNA synthetase
28	*GUK1*	2.58e-05	5.61	guanylate kinase 1
29	*RPS5*	2.58e-05	4.66	ribosomal protein S5
30	*MTL5*	3.3e-05	14.60	metallothionein-like 5, testis-specific (tesmin)
31	MIF *	3.8e-05	10.72	macrophage migration inhibitory factor (glycosylation-inhibiting factor)
32	*POLR2H*	3.94e-05	4.68	polymerase (RNA) II (DNA directed) polypeptide H
33	*IFITM2*	4.38e-05	4.17	interferon induced transmembrane protein 2 (1–8D)
34	*MUTYH*	4.41e-05	19.61	mutY homolog (E. coli)
35	*ITPKA*	4.41e-05	15.19	inositol 1,4,5-trisphosphate 3-kinase A
36	*FDPS*	5.36e-05	3.45	farnesyl diphosphate synthase
37	*HIST1H2BG*	6.08e-05	10.23	histone 1, H2bg
38	*INPP5D*	6.15e-05	17.02	inositol polyphosphate-5-phosphatase, 145 kDa
39	*IDH3G*	6.47e-05	47.05	isocitrate dehydrogenase 3 (NAD+) gamma
40	*ERF*	6.49e-05	15.57	Ets2 repressor factor
41	*PTPRJ*	6.54e-05	13.86	protein tyrosine phosphatase, receptor type, J
42	*ATP5G1*	7.90e-05	4.82	ATP synthase, H+ transporting, mitochondrial F0 complex, subunit c, isoform 1
43	*CDC45L*	8.49e-05	22.94	CDC45 cell division cycle 45-like (S. cerevisiae)
44	*ENO1*	9.67e-05	4.75	enolase 1, (alpha)
45	*IDH3B*	0.0001064	3.81	isocitrate dehydrogenase 3 (NAD+) beta
46	*ST3GAL4*	0.0001140	28.99	ST3 beta-galactoside alpha-2,3-sialyltransferase 4
47	*DDR2*	0.0001152	15.87	discoidin domain receptor family, member 2
48	*IFI30*	0.0001188	3.88	interferon, gamma-inducible protein 30
49	*EIF4G1*	0.0001282	3.31	eukaryotic translation initiation factor 4 gamma, 1
50	*CXorf27*	0.0001372	11.62	chromosome X open reading frame 27
51	*EPB41*	0.0001423	4.05	erythrocyte membrane protein band 4.1 (elliptocytosis 1, RH-linked)
52	*RPS15*	0.0001427	6.91	ribosomal protein S15
53	*ABCA3*	0.0001432	30.18	ATP-binding cassette, sub-family A (ABC1), member 3
54	*CGA*	0.0001508	12.31	glycoprotein hormones, alpha polypeptide
55	*SLC37A4*	0.0001527	23.08	solute carrier family 37 (glycerol-6-phosphate transporter), member 4
56	*NDUFB8*	0.0001527	3.56	NADH dehydrogenase (ubiquinone) 1 beta subcomplex, 8, 19 kDa
57	*CSPG4*	0.0001583	60.14	chondroitin sulfate proteoglycan 4 (melanoma-associated)
58	*MTHFD1*	0.0001583	21.57	methylenetetrahydrofolate dehydrogenase (NADP+ dependent) 1,
59	*CYP3A5*	0.0001586	17.90	cytochrome P450, family 3, subfamily A, polypeptide 5
60	*FKBP4*	0.0001611	6.51	FK506 binding protein 4, 59 kDa
61	*SCO2*	0.0001760	14.31	SCO cytochrome oxidase deficient homolog 2 (yeast)
62	*DPM2*	0.0001846	12.99	dolichyl-phosphate mannosyltransferase polypeptide 2, regulatory subunit
63	*NRGN*	0.0001945	16.26	neurogranin (protein kinase C substrate, RC3)
64	*TUBA2*	0.0001988	8.87	tubulin, alpha 2
65	*GAPD*	0.0001990	4.99	glyceraldehyde-3-phosphate dehydrogenase
66	*CDK4*	0.0002205	6.73	cyclin-dependent kinase 4
67	*BIRC5*	0.0002380	6.62	baculoviral IAP repeat-containing 5 (survivin)
68	LAMB3 *	0.0002380	5.33	laminin, beta 3
69	*SSR4*	0.0002380	3.81	signal sequence receptor, delta (translocon-associated protein delta)
70	*PIK4CA*	0.0002398	12.01	phosphatidylinositol 4-kinase, catalytic, alpha polypeptide

* Genes appeared in table 4

**Table 3:** Top 70 down-regulated genes

**No**.	**Gene symbol**	**False discovery rate**	**Fold difference**	**Description**
1	*HERC3*	< 1e-07	0.10	hect domain and RLD 3
2	*PDCD4*	< 1e-07	0.10	programmed cell death 4 (neoplastic transformation inhibitor)
3	*SFRS5*	< 1e-07	0.11	splicing factor, arginine/serine-rich 5
4	*ST6GAL1*	< 1e-07	0.13	ST6 beta-galactosamide alpha-2,6-sialyltranferase 1
5	*DXYS155E*	< 1e-07	0.16	DNA segment on chromosome X and Y (unique) 155 expressed sequence
6	*KIAA1641*	< 1e-07	0.17	KIAA1641
7	*LEPROTL1*	< 1e-07	0.17	leptin receptor overlapping transcript-like 1
8	*ELL2*	< 1e-07	0.23	elongation factor, RNA polymerase II, 2
9	*FUSIP1*	< 1e-07	0.24	FUS interacting protein (serine-arginine rich) 1
10	*OGT*	< 1e-07	0.24	O-linked N-acetylglucosamine (GlcNAc) transferase
11	*MYST4*	< 1e-07	0.26	MYST histone acetyltransferase (monocytic leukemia) 4
12	*RUVBL2*	0.0000063	0.13	RuvB-like 2 (E. coli)
13	*ARF6*	0.0000063	0.13	ADP-ribosylation factor 6
14	*ACTR2*	0.0000063	0.14	ARP2 actin-related protein 2 homolog (yeast)
15	*TRIM23*	0.0000063	0.15	tripartite motif-containing 23
16	*HDAC3*	0.0000063	0.15	histone deacetylase 3
17	*PRKAR1A*	0.0000063	0.17	protein kinase, cAMP-dependent, regulatory, type I, alpha
18	*DR1*	0.0000063	0.18	down-regulator of transcription 1, TBP-binding (negative cofactor 2)
19	*MAP4K3*	0.0000063	0.21	mitogen-activated protein kinase kinase kinase kinase 3
20	*PPP1CB*	0.0000063	0.21	protein phosphatase 1, catalytic subunit, beta isoform
21	*SEL1L*	0.0000063	0.24	sel-1 suppressor of lin-12-like (C. elegans)
22	*RAD17*	0.0000063	0.24	RAD17 homolog (S. pombe)
23	*ABI1*	0.0000063	0.27	abl-interactor 1
24	*ATM*	0.0000063	0.27	ataxia telangiectasia mutated (includes complementation groups A, C and D)
25	*STK17A*	0.0000063	0.28	serine/threonine kinase 17a (apoptosis-inducing)
26	*PRR4*	0.0000094	0.02	proline rich 4 (lacrimal)
27	*CHL1*	0.0000094	0.06	cell adhesion molecule with homology to L1CAM (close homolog of L1)
28	*PRKACB*	0.0000094	0.10	protein kinase, cAMP-dependent, catalytic, beta
29	*ELF1*	0.0000094	0.13	E74-like factor 1 (ets domain transcription factor)
30	*EPS15*	0.0000094	0.16	epidermal growth factor receptor pathway substrate 15
31	*NKTR*	0.0000094	0.20	natural killer-tumor recognition sequence
32	*DNAJC10*	0.0000094	0.21	DnaJ (Hsp40) homolog, subfamily C, member 10
33	*RARB*	0.0000094	0.25	retinoic acid receptor, beta
34	*FBXO9*	0.0000094	0.29	F-box protein 9
35	*MSMBROCK1*	0.0000120	0.13	microseminoprotein, beta-
36	*TADA3L*	0.0000120	0.14	Rho-associated, coiled-coil containing protein kinase 1
37	*TNFAIP8*	0.0000120	0.16	transcriptional adaptor 3 (NGG1 homolog, yeast)-like
38	*TGFBR2*	0.0000120	0.16	tumor necrosis factor, alpha-induced protein 8
39	*SLC23A1*	0.0000120	0.19	transforming growth factor, beta receptor II (70/80 kDa)
40	*FOXO1A*	0.0000120	0.20	solute carrier family 23 (nucleobase transporters), member 1
41	*USP12*	0.0000120	0.20	forkhead box O1A (rhabdomyosarcoma)
42	*CD2AP*	0.0000134	0.18	ubiquitin specific protease 12
43	*UBE4A*	0.0000134	0.18	CD2-associated protein
44	*SCP2*	0.0000134	0.21	ubiquitination factor E4A (UFD2 homolog, yeast)
45	*CAPN7*	0.0000134	0.23	sterol carrier protein 2
46	*PTPRS*	0.0000134	0.24	calpain 7
47	*BCL6*	0.0000134	0.25	protein tyrosine phosphatase, receptor type, S
48	*AGA*	0.0000134	0.32	B-cell CLL/lymphoma 6 (zinc finger protein 51)
49	*PROS1*	0.0000134	0.33	aspartylglucosaminidase
50	*UBL3*	0.0000155	0.06	protein S (alpha)
51	*BRF1*	0.0000155	0.12	ubiquitin-like 3
52	*ANP32B*	0.0000155	0.15	BRF1 homolog, subunit of RNA polymerase III transcription initiation factor IIIB
53	*AMY2B*	0.0000155	0.25	acidic (leucine-rich) nuclear phosphoprotein 32 family, member B
54	*FUBP3*	0.0000167	0.07	amylase, alpha 2B; pancreatic
55	*MAN2A1*	0.0000167	0.15	far upstream element (FUSE) binding protein 3
56	*TERF2IP*	0.0000167	0.21	mannosidase, alpha, class 2A, member 1
57	*PRKRIR*	0.0000167	0.24	telomeric repeat binding factor 2, interacting protein
58	*HRB2*	0.0000167	0.27	protein-kinase, interferon-inducible double stranded RNA dependent inhibitor, repressor of (P58 repressor)
59	*MAP4K5*	0.0000167	0.28	HIV-1 rev binding protein 2
60	*PPM1B*	0.0000184	0.16	mitogen-activated protein kinase kinase kinase kinase 5
61	*PSIP1*	0.0000184	0.16	protein phosphatase 1B (formerly 2C), magnesium-dependent, beta isoform
62	*TMEM1*	0.0000184	0.18	PC4 and SFRS1 interacting protein 1
63	*CDK5R1*	0.0000184	0.31	transmembrane protein 1
64	*ZA20D2*	0.0000203	0.27	cyclin-dependent kinase 5, regulatory subunit 1 (p35)
65	*AIM2*	0.0000216	0.14	zinc finger, A20 domain containing 2
66	*NBR1*	0.0000216	0.19	absent in melanoma 2
67	*TCN1*	0.0000216	0.26	neighbor of BRCA1 gene 1
68	*STXBP3*	0.0000225	0.20	transcobalamin I (vitamin B12 binding protein, R binder family)
69	*ZNF265*	0.0000225	0.20	syntaxin binding protein 3
70	*HERC3*	0.0000240	0.24	zinc finger protein 265

### Proposed biomarkers and known oncogenes/tumor suppression genes for NPC

In 2006, we proposed common cancer biomarkers expressed in cancers of various histology (melanoma, colon, ovarian, and esophageal carcinoma) but not in normal tissues. The analysis included training and a prediction set and only genes highly significantly specific for the cancerous tissues were proposed as biomarker candidates. 16 genes were proposed as common cancer biomarkers based on 20 cDNA clones (cutoff P < 1.7 × 10^-16^), showing a prediction accuracy of approximately 90% [16]. To verify whether the previous results were predictive also for NPC, a comparison was made between the two studies; 6 genes (CYC1, MIF, LAMB3, TSTA3, TUBB2, and UBE2C) of the 16 genes proposed by the previous study were consistently highly expressed in NPC as well. The seventh, TRAP1, was also included among the first 50 genes in the previous study (Table 4). In addition, CyC1, MIF, and LAMB3 appeared among the top 70 up-regulated genes in NPC (Table 2).

**Table 4:** Proposed NPC biomarkers according to our previous study (Basil et al [14])

**Gene symbol**	**Fold difference (NPC/NP)**	**Parametric p-value**	**False discovery rate**	**Description**
CYC1 *	10.36	4.00E-07	1.34E-05	Cytochrome c-1
MIF *	10.72	2.10E-06	3.83E-05	Macrophage migration inhibitory factor
LAMB3 *	5.33	2.43E-05	0.000238	Laminin, beta 3
TSTA3	27.22	9.77E-05	0.0007454	Tissue specific transplantation antigen P35B
TUBB2	43.51	0.0001256	0.0009051	Tubulin, beta 2
UBE2C	20.29	0.0009964	0.0045488	Ubiquitin-conjugating enzyme E2C
TRAP1	10.94	0.0026581	0.0097038	TNF receptor-associated protein 1

* The genes were in top 70 up-regulated genes of table 2

MILANO analysis utilizes archived literature as a database linking gene name with reported biological functions. To detect whether these 692 differentially expressed genes were known to be associated with NPC oncogenesis and tumor progression, the following key words: oncogene, proto-oncogene, tumor suppressor and nasopharyngeal carcinoma were applied as input in the MILANO searcher engine. In this way, we could screen for known and candidate oncogenes or tumor suppressor genes reported in other human malignancies or in NPC. Table 5 presents 10 genes (4 up-regulated and 6 down-regulated in NPC) with known oncogenic or tumor suppressor function in various human malignancies. Some genes, such as MIF and CCDN2, bore predominant pro-tumor progression functions, other genes, such as KLF5 and TGFBR2, contribute to tumor suppression functions. Notably, the results from MILANO analysis were consistent with our microarray data; the differential expression of the genes proposed as oncogene or Proto-oncogene by MILANO consisted of genes with >5-fold difference (our microarray data), ranging from 6.62 to 20.15. Conversely, the fold difference of genes associated with tumor suppressor function by MILANO was less than 0.5, ranging from 0.14 to 0.27.

**Table 5:** MILANO analysis for oncogenes and tumor suppressor genes

**Gene symbol**	**Role^1^**	**Fold difference^2 ^(NPC/NP)**	**Description**	**Map Location**
MIF	Candidate oncogene	10.72	macrophage migration inhibitory factor (glycosylation-inhibiting factor)	22q11.23
BIRC5	Oncogene	6.62	baculoviral IAP repeat-containing 5 (survivin)	17q25
PTTG1	Proto-oncogene	7.35	pituitary tumor-transforming 1	5q35.1
CCND2	Proto-oncogene	20.15	cyclin D2	12p13
ATM	Tumor suppressor	0.27	ataxia telangiectasia mutated (includes complementation groups A, C and D)	11q22-q23
FOXO1A	Tumor suppressor	0.20	forkhead box O1A (rhabdomyosarcoma)	13q14.1
TGFBR2	Tumor suppressor	0.19	transforming growth factor, beta receptor II	3p22
PRKAR1A	Tumor suppressor	0.17	protein kinase, cAMP-dependent, regulatory, type I, alpha	17q23-q24
KLF5	Tumor suppressor	0.17	Kruppel-like factor 5 (intestinal)	13q22.1
PDCD4	Tumor suppressor	0.10	programmed cell death 4 (neoplastic transformation inhibitor)	10q24

^1 ^Role: functions identified by MILANO

^2 ^Fold difference: extracted from our microarray data.

### Computational pathway analysis

The pathway analysis was done using the gene set expression comparison kit [11] implemented in BRB-ArrayTools. The human pathway lists determined by KEGG Pathways Database was selected. Significance threshold of Hotelling's T-square test was set at 0.005 using the first three principal components. Significant pathways were listed in Table 6. Among them, several pathways were involved in cell growth and death, important signal transduction and immune system. Interestingly, several pathways associated with immune function are involved (Table 6 and 7) with the overexpression of interleukin-1β and interleukin-6.

**Table 6:** Significant pathways at the nominal 0.005 level of the Hotelling T-square test *

**Related Function**	**Kegg Pathway**	**Pathway description**	**Number of genes**	**p-value**
Cell Growth & Death	hsa04210	Apoptosis	9	1.20E-06
	hsa04110	Cell cycle	10	2.10E-06
Signal Transduction	hsa04020	Calcium signaling pathway	13	1.80E-06
	hsa04010	MAPK signaling pathway	19	1.11E-05
	hsa04310	Wnt signaling pathway	10	1.24E-05
	hsa04630	Jak-STAT signaling pathway	7	3.64E-05
	hsa04070	Phosphatidylinositol signaling system	7	8.03E-05
	hsa04370	VEGF signaling pathway	5	0.000333
Immune System	hsa04650	Natural killer cell mediated cytotoxicity	7	1.67E-05
	hsa04640	Hematopoietic cell lineage	6	2.63E-05
	hsa04662	B cell receptor signaling pathway	9	5.01E-05
	hsa04660	T cell receptor signaling pathway	6	5.06E-05
	hsa04664	Fc epsilon RI signaling pathway	7	9.76E-05
	hsa04610	Complement and coagulation cascades	5	0.0001297
	hsa04620	Toll-like receptor signaling pathway	5	0.003536
Signaling Molecules and Interaction	hsa04080	Neuroactive ligand-receptor interaction	12	1.00E-07
	hsa04060	Cytokine-cytokine receptor interaction	15	3.00E-07
	hsa04514	Cell adhesion molecules (CAMs)	9	1.50E-05
	hsa04512	ECM-receptor interaction	8	0.0004294
Endocrine System	hsa04910	Insulin signaling pathway	8	6.00E-06
	hsa04920	Adipocytokine signaling pathway	5	6.90E-06
	hsa03320	PPAR signaling pathway	6	3.42E-05
	hsa04912	GnRH signaling pathway	8	3.66E-05
Cell Communication	hsa04510	Focal adhesion	17	9.20E-06
	hsa04520	Adherens junction	5	2.95E-05
	hsa04540	Gap junction	10	3.89E-05
	hsa04530	Tight junction	10	4.48E-05
Others	hsa04810	Regulation of actin cytoskeleton	11	3.90E-06
	hsa00260	Glycine, serine and threonine metabolism	6	1.54E-05
	hsa00190	Oxidative phosphorylation	17	3.14E-05
	hsa00230	Purine metabolism	18	3.22E-05
	hsa00500	Starch and sucrose metabolism	9	7.68E-05
	hsa00980	Metabolism of xenobiotics by cytochrome P450	7	8.06E-05
	hsa00240	Pyrimidine metabolism	12	0.0001352
	hsa04730	Long-term depression	6	0.0002265
	hsa04720	Long-term potentiation	7	0.0003216
	hsa04360	Axon guidance	11	0.0007991

* Significance threshold of permutation tests: 0.005

Significance threshold of Hotelling's T-square tests: 0.005

Number of principal components for Hotelling's T-square tests: 3

Square tests: 3

**Table 7:** List of genes involving in Immune function

**Pathway**	**Description**	**Gene symbol**	**Parametric p-value**	**Fold Difference (NPC/NP)**
	protein kinase C, beta 1	PRKCB1	2.20E-05	0.14
	integrin, beta 2 (antigen CD18 (p95), lymphocyte function-associated antigen 1; macrophage antigen 1 (mac-1) beta subunit)	ITGB2	0.0001733	0.18
Natural killer cell mediated	nuclear factor of activated T-cells, cytoplasmic, calcineurin-dependent 1	NFATC1	0.0004205	0.23
cytotoxicity	phosphoinositide-3-kinase, catalytic, gamma polypeptide	PIK3CG	0.0002216	0.24
	vav 2 oncogene	VAV2	0.0024579	7.77
	TYRO protein tyrosine kinase binding protein	TYROBP	0.0005303	14.39
	tumor necrosis factor receptor superfamily, member 10b	TNFRSF10B	0.0006507	14.91
	complement component (3d/Epstein Barr virus) receptor 2	CR2	0.0001842	0.02
	interleukin 1, beta	IL1B	0.0043926	8.27
Hematopoietic cell lineage	interleukin 6 (interferon, beta 2)	IL6	0.0024281	8.29
	integrin, beta 3 (platelet glycoprotein IIIa, antigen CD61)	ITGB3	0.0008031	10.39
	CD14 antigen	CD14	4.15E-05	16.88
	colony stimulating factor 1 (macrophage)	CSF1	1.00E-06	20.35
	complement component (3d/Epstein Barr virus) receptor 2	CR2	0.0001842	0.02
	protein kinase C, beta 1	PRKCB1	2.20E-05	0.14
B cell receptor	Bruton agammaglobulinemia tyrosine kinase	BTK	5.39E-05	0.17
signaling pathway	nuclear factor of activated T-cells, cytoplasmic, calcineurin-dependent 1	NFATC1	0.0004205	0.23
	phosphoinositide-3-kinase, catalytic, gamma polypeptide	PIK3CG	0.0002216	0.24
	nuclear factor of kappa light polypeptide gene enhancer in B-cells 2	NFKB2	0.0019236	7.57
	vav 2 oncogene	VAV2	0.0024579	7.77
	glycogen synthase kinase 3 beta	GSK3B	0.0021047	13.20
	inositol polyphosphate-5-phosphatase, 145kDa	INPP5D	4.10E-06	17.02
	nuclear factor of activated T-cells, cytoplasmic, calcineurin-dependent 1	NFATC1	0.0004205	0.23
	phosphoinositide-3-kinase, catalytic, gamma polypeptide	PIK3CG	0.0002216	0.24
T cell receptor	cyclin-dependent kinase 4	CDK4	2.20E-05	6.73
signaling pathway	nuclear factor of kappa light polypeptide gene enhancer in B-cells 2	NFKB2	0.0019236	7.57
	vav 2 oncogene	VAV2	0.0024579	7.77
	mitogen-activated protein kinase kinase kinase 14	MAP3K14	0.0008295	11.17
	membrane-spanning 4-domains, subfamily A, member 2 (Fc fragment of IgE, high affinity I, receptor for; beta polypeptide)	MS4A2	6.30E-06	0.04
Fc epsilon RI signaling pathway	protein kinase C, beta 1	PRKCB1	2.20E-05	0.14
	Bruton agammaglobulinemia tyrosine kinase	BTK	5.39E-05	0.17
	phosphoinositide-3-kinase, catalytic, gamma polypeptide	PIK3CG	0.0002216	0.24
	phospholipase A2, group V	PLA2G5	0.0017931	6.86
	vav 2 oncogene	VAV2	0.0024579	7.77
	inositol polyphosphate-5-phosphatase, 145kDa	INPP5D	4.10E-06	17.02
	complement component 3	C3	2.25E-05	0.01
Complement and coagulation	complement component (3d/Epstein Barr virus) receptor 2	CR2	0.0001842	0.02
cascades	protein S (alpha)	PROS1	5.00E-07	0.06
	coagulation factor XIII, A1 polypeptide	F13A1	0.0002893	0.22
	complement factor H	CFH	0.0018395	0.26
	phosphoinositide-3-kinase, catalytic, gamma polypeptide	PIK3CG	0.0002216	0.24
Toll-like receptor signaling pathway	nuclear factor of kappa light polypeptide gene enhancer in B-cells 2	NFKB2	0.0019236	7.57
	interleukin 1, beta	IL1B	0.0043926	8.27
	interleukin 6 (interferon, beta 2)	IL6	0.0024281	8.29
	CD14 antigen	CD14	4.15E-05	16.88

### Validation of differential regulated genes by Semi-quantitative RT-PCR and Immunohistochemistry

**Figure 2:**
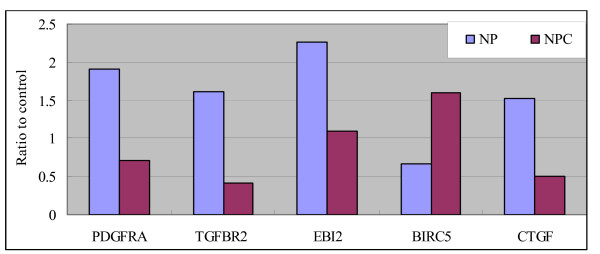
Histogram identification of differentially expressed genes using semi-quantitative RT-PCR (average ratio).

To validate the microarray data, differential gene expression was confirmed by Semi-quantitative RT-PCR in 5 genes randomly selected from the 692 differentially expressed genes. These genes displayed similar expression patterns (Figure 2) to the microarray data; ratio of expression in NPC/NP was 0.32 in CTGF, 0.24 in TGFBR2, 0.36 in PDGFRA, 0.46 in EBI2, 2.67 in BIRC5, which was concordant with array data.

**Figure 3:**
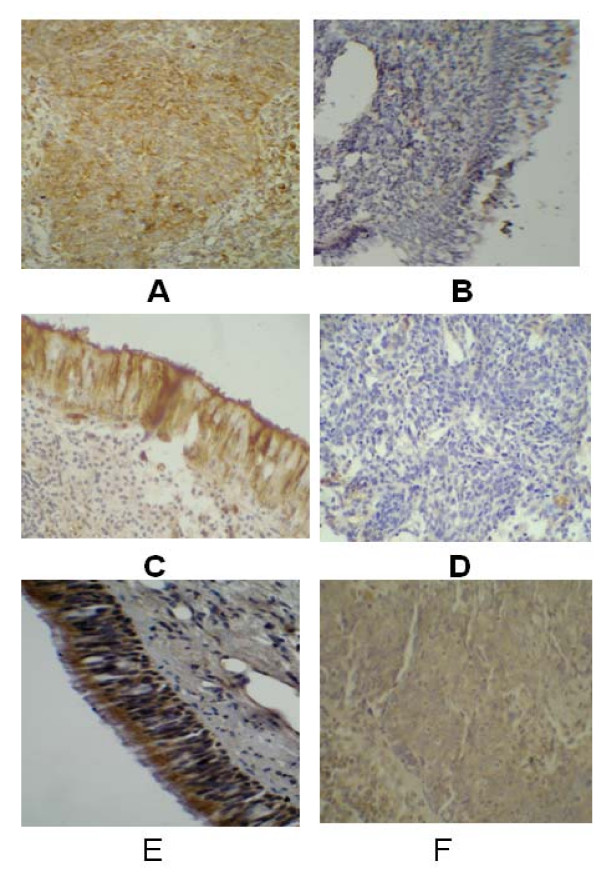
**Immunohistochemistry detection of three proteins in NPC and NP (×200)**. A: BIRC5 in NPC (strong positive); B: BIRC5 in NP (weak positive); C: CTGF in NP (strong positive); D: CTGF in NPC (weak positive); E: TGFBR2 in NP (strong positive); F: TGFBR2 in NPC (weak positive).

To further evaluate the reliability of the microarray data, three genes, selected as representatives were analyzed by Immunohistochemistry. According to our array analysis, BIRC5 was up-regulated and both of TGFBR2 and CTGF(Figure 3) were down-regulated in NPC. The protein expression of these three genes was observed in totally 55 NPC samples and 46 NP samples. The Mann-Whitney test of positive rates in NPC and NP samples indicated that BIRC5 protein expression was significantly higher in NPC (P < 0.004) compared to NP samples, while TGFBR2 and CTGF were significantly lower in NPC (P < 0.002 and 0.001 respectively) compared to NP samples, supporting the reliability of the array data.

## Discussion

Nasopharyngeal carcinoma is a special type of squamous cell carcinoma of head and neck associated with EBV infection, environmental factors and genetic aberrance. Clinically, poorly differentiated squamous cell carcinoma may account for 98% NPC patients while well differentiated squamous cell carcinoma and adenocarcinoma are rarely encountered in Southern China where this disease is particularly prevalent. The present study focused on alteration of gene expression in poorly differentiated squamous cell carcinoma of the nasopharynx. Owing to anatomical limitations, NPC and NP specimens collected from the clinic are usually very scarce and, because in most cases biopsies are semi-blindly performed through a forceps rather than a scalpel, it, is hard to accurately exclude normal NP tissue contamination from cancerous ones. Even in NPC biopsies without normal NP cells, the content of NPC cell appears to vary greatly. Therefore, screening the specimens obtained from the clinic for presence of a sufficient number of cancer cells was the critical step in order to assure the uniformity and validity of the microarray results. We found that tumor cell content varied from 5% to 95% in 102 pathologically diagnosed primary NPC samples among which only 32 samples contained more than 70% of cancer cells; the latter were qualified for further analysis.

In the present study, we applied the cDNA microarray technology to study differential gene expression between NPC and NP samples from Southern China. A pooling strategy was used to improve the statistical power and reduce individual heterogeneity. Class comparison analysis revealed 692 deferentially expressed genes with 2-fold or greater difference at a significance p-value of < 0.005. Of these genes, 435 were up-regulated and 257 were down-regulated. Subsequently, all the differentially expressed genes were further screened by MILANO analysis. After excluding the genes without association with limited oncogene and tumor suppressor terms when searching in MILANO, 10 genes were found to be reported consistently as known oncogenes and tumor suppressor genes in various human malignancies in previous studies. 4 genes were up-regulated and 6 genes down-regulated. Interestedly, of these 10 genes, three genes (MIF, BIRC5 and ATM) have been documented in NPC associated articles, and the rest genes (PTTG1, FOXO1A, TGFBR2, PRKAR1A, CCND2, KLF5, and PDCD4) have never reported or poorly understood in NPC before.

It is well known that oncogenes tend to be up-regulated and tumor suppressor genes are down-regulated in malignant condition. Based on CGH, chromosomal abnormalities associated with primary and metastatic NPC have been identified in all human chromosomes except chromosome Y [3,4,7,17-20]. BIRC5 up-regulated in NPC (oncogenes) was located on 17q21.3-25. As down-regulated tumor suppressors in NPC, FOXO1A and KLF5 existed on 13q14.1-34, ATM on 11 q13-23, and TGFBR2 on 3p21.3-24. The expression level of these genes was consistent with corresponding chromosomal gains or losses. However, the gene expression of the rest of the genes did not correspond with their chromosomal status. MIF and PTTG1 up-regulated in NPC (oncogenes) were on 19p13.3 and 5q35.1 respectively and PRKAR1A and PDCD4 down-regulated in NPC (tumor suppressors) were mapped to chromosome 17q22-q24 and 10q24 respectively, but no corresponding high frequency of chromosomal alterations in those region have been reported in association with NPC. Down-regulation of some genes might be involved in other mechanisms including promoter hypermethylation or additional modification, promoter mutation and gene mutation.

Several studies have shown that several differentially expressed genes identified in this study have functions associated with cell proliferation, cell apoptosis, cell cycle, angiogenesis and signal transducer activity, creating an active proliferation and progression status at tumor site. As an up-regulated oncogene, MIF, a macrophage migration inhibitory factor, may function as an autocrine mediator of both growth factor- and integrin-dependent sustained ERK MAPK activation, cyclin D1 expression, and cell cycle progression. Swant[19] found that MIF could stimulate cyclin D1 by recruiting Rho GTPase and its downstream signaling for MAPK activation. EBV oncogene LMP1 in NPC, PTGS2 in endometrial carcinoma[21] and MYC in breast cancer[22] may induce the BIRC5 expression by different pathways. As a tumor suppressor, KLF5, a transcription factor associated with cellular signaling involved in cell proliferation and oncogenesis, inhibits the expression of BIRC5 by binding p53 in acute lymphoblastic leukemia[23], suggesting that anti-apoptotic factor BIRC5 may play a key role in gene-regulated network of NPC. Pituitary tumor-transforming gene-1 (PTTG1) is overexpressed in a variety of endocrine-related tumors and nonendocrine-related cancers involving the central nervous, pulmonary, and gastrointestinal systems. It is a potent oncogene because of its ability to combine with p53 and thus to prevent p53 from binding to DNA and inducing cell death[24]. DNA damage and the consequent activation of the DNA damage response (DDR) pathway is one of the occasions for the tumor initiation and progression. Among tumor suppressors, ATM, a major regulator of the cellular response to DNA double-strand breaks, may be a key factor in the DDR pathway. Knocking down ATM with a short hairpin RNA (shRNA) could block p53 induction in response to aberrant STAT5A activation and bypass the senescence response to this oncogene when the Rb pathway was also inactivated. In addition, knocking down ATM could inhibit E2F1-induced senescence and, in combination with Rb inactivation, suppress RasV12-induced senescence[25]. TGF-β exerted its tumor-suppressor effects on many tumors by binding to the transmembrane TGF-β type II receptor (TGFBR2, a tumor suppressor), which caused the recruitment of the TGF-β type I receptor (TGFBR1) with subsequent activation of the receptor complex. Decreased expression of TGFBR2 found in NPC may block this important pathway of tumor-growth inhibition and promote the NPC malignant transformation[26]. Programmed cell death-4 (PDCD4) is a new discovered tumor suppressor protein that inhibits protein synthesis by suppression of translation initiation. A recent study showed that PDCD4 suppressed tumor progression in human colon carcinoma cells by the novel mechanism of down-regulating MAP4K1 transcription, with consequent inhibition of c-Jun activation and AP-1-dependent transcription[27]. Down-regulated expression of PDCD4 in NPC tissue and cells suggested a negatively regulation role in NPC pathogenesis. Tumor suppressor FOXO1A, a FOXO transcription factor, has been implicated in several human cancers. However, it has not been reported in NPC to date. Shore[28] found that Expression of the EBV genes for latent membrane protein 1 and latent membrane protein 2A could decrease Foxo1A expression by phosphatidylinositol 3-kinase-mediated nuclear export. Oncoprotein P3k and Akt can suppress the FoxO1 expression by a common denominator of their pathway[29]. The tumor suppressor gene, PRKAR1A, coding for the Type 1alpha regulatory subunit of protein kinase A, a critical cellular component of a number of cyclic nucleotide-dependent signaling pathways, is mutated in Carney complex, a familial neoplasia syndrome that is associated with thyroid tumors[30]. Its decreased expression in NPC may be caused by similar gene mutation, which is remained to be further elucidated.

Interestedly, several studies really supported the dysregulation of several pathways, such as Wnt signaling, MARK signaling [31,32], NF-kB-Apoptosis resistance, integrin signaling[32] in NPC although most of pathways involving cell growth and death, cell communication, and immune system, etc. have not been well documented. In our present study, our computational pathway analysis of 692 differentially expressed genes strongly supported that multiple biological pathways were indeed involved in NPC oncogenesis. Particularly, we found that most of immune system associated pathways, such as Natural killer cell mediated cytotoxicity, Hematopoietic cell lineage, B cell receptor signaling, T cell receptor signaling, Fc epsilon RI signaling, Complement and coagulation cascades, and Toll-like receptor signaling, were involved, suggesting that they may play important roles in NPC.

Recently, there has been increasing interest in identify diagnostic tools that could complement standard histopathologic evaluation to determine the presence of cancer cells in tissues[33], a previous study from our group applied a high throughput microarray technology to investigate a broad range of cancer types and identified 16 proposed universal cancer biomarkers with high prediction accuracy. These biomarkers could be used broadly to improve the sensitivity and specificity of cancer staging and early detection of loco-regional or systemic recurrence [16]. Among the 16 common cancer biomarkers identified as highly predictive by the previous study which did not include NPC samples, 6 genes (CYC1, MIF, LAMB3, TSTA3, TUBB2, and UBE2C) were found to be highly expressed in NPC by the study. The seventh gene, TRAP1, was also included in the first 50 genes. Therefore, CYC1, MIF, LAMB3, TSTA3, TUBB2, UBE2C, and TRAP1 may possibly represent NPC candidate biomarkers adding a molecular dimension to the histo-pathological diagnosis of the disease and these biomarkers may be added to the pathologist's repertoire for the uncovering of NPC pathogenesis when comprehensive histologic evaluation is not sufficient. Obviously further validation will be needed in the future. Moreover, NPC could share the expression of various biomarkers with other cancers of disparate histology suggesting the existence of common pathways of oncogenesis that may be most relevant as therapeutic targets.

## Conclusion

Using microarray technology and bioinformatics analysis, we identified 692 differentially expressed genes. Among these genes, 10 known oncogenes and tumor suppressors were picked out as key genes which mainly promoted NPC tumor genesis. Seven genes were also proposed as NPC candidate cancer biomarkers as they were observed universal cancer biomarkers in a previous study analyzing tumors of different histology. Computational pathway analysis suggested the likelihood that the multi-pathways are involved in the oncogenesis of NPC including a chronic inflammatory process mediated through classic cytokine pathways.

## Competing interests

The authors declare that they have no competing interests.

## Authors' contributions

All authors have read and approved the final manuscript, WF set up the protocols, WF,XL,QJ and ZL contributed in the experimental procedures and in the interpretation of the data, HY,SW,SX,QL,TL,JH,WX,ZL and YZ gave advises on the work and helped in the interpretation of the data, FMM and KY supervised all the work and wrote the paper together with WF,XL.
